# The Role of Vitamin D and Vitamin D Receptor in Sepsis

**DOI:** 10.3390/cimb47070500

**Published:** 2025-07-01

**Authors:** Shenglan Shang, Dongxin Chen, Yuqi Wei, Shuo Zou, Qiuyue Chang, Hong Zhou, Airong Yu

**Affiliations:** 1 Department of Clinical Pharmacy, General Hospital of Central Theater Command, Wuhan 430070, China; s_shang0818@163.com (S.S.); dongxinchen1990@163.com (D.C.); weiyuqi7745@163.com (Y.W.); sojaoad777@outlook.com (S.Z.); cmqy1739@163.com (Q.C.); 2 Key Laboratory of Basic Pharmacology of Ministry of Education and Joint International Research Laboratory of Ethnomedicine of Ministry of Education, Zunyi Medical University, Zunyi 563006, China

**Keywords:** vitamin D, vitamin D receptor, sepsis, immunomodulation, anti-inflammation

## Abstract

Sepsis acts as the leading cause of mortality in intensive care units, characterized by life-threatening organ dysfunction due to a dysregulated host response to infection. Vitamin D (VD) pleiotropic functions were demonstrated in different biological processes, including inflammation and immunity. VD receptor (VDR) is a member of the nuclear receptor superfamily, involved in immunoregulation and resistance to infections. Previous studies have demonstrated that VD deficiency is a potential risk factor for sepsis development, which may be regulated by VDR-related physiological processes. In this review, we present a comprehensive overview of the roles of VD and VDR in sepsis, focusing on immune modulation, anti-inflammatory and anti-infective responses, oxidative stress regulation, gut microbiome enhancement, vascular endothelial cell modulation, and antiplatelet activity. We also discuss recent advances in clinical research on VD/VDR in sepsis, considering the clinical implications and potential interventions of VD analogs and VDR ligands in treatment. Despite its challenges, VD holds potential for personalized sepsis interventions. Additionally, VD/VDR may serve as a promising bidirectional immunomodulator, capable of addressing both hyperinflammatory and immunosuppressive phases of sepsis, yet require systematic investigations into its dynamic states and functions across different sepsis phases. Ongoing study and evidence-based guidelines are crucial to maximize its therapeutic benefits and improve clinical outcomes.

## 1. Introduction

Sepsis is a life-threatening organ dysfunction caused by a dysregulated host response to infection. Further development of sepsis leads to septic shock and multiple organ dysfunction syndrome (MODS), which is a common complication in critically ill patients suffering from trauma/burns, infections, and shock [1]. Sepsis remains a leading cause of mortality in patients in intensive care units (ICUs) worldwide [2]. A recent analysis from the Global Burden of Disease Study reported 48.9 million annual cases of sepsis worldwide, with 11 million related deaths in 2017 [3]. Sepsis still contributes to almost 20% of all deaths every year in the world, more than 20 deaths every minute [4,5]. Furthermore, a recent meta-analysis indicated a mortality rate of 26.7% in sepsis patients, highlighting a 46% rise in incidence since 2008 [6]. However, the mechanisms underlying sepsis remain incompletely understood, potentially involving dysregulation of inflammatory responses, immune dysfunction, and coagulation disorder. Currently, the clinical management of sepsis typically includes standard approaches such as infection control, fluid resuscitation, inflammation suppression, and immune modulation, yet there is a notable lack of specific therapeutic agents targeting sepsis.

**Figure 1:**
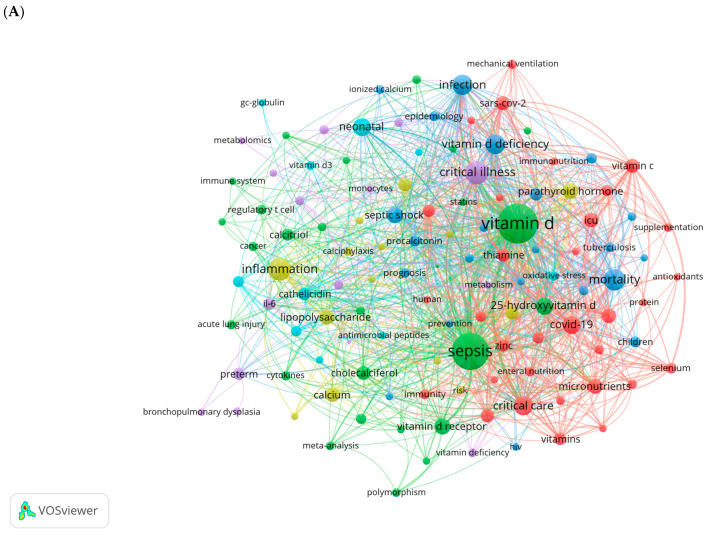

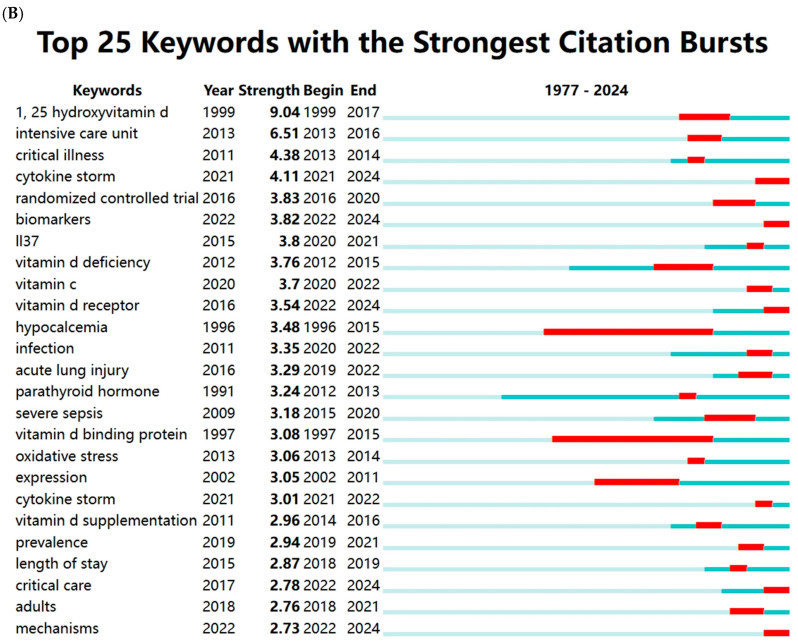
Keywords cluster analysis. (**A**) Co-occurrence visualization network of keywords using VOSviewer (1.6.20). (**B**) Visualization map of top 25 keywords with the strongest citation bursts using CiteSpace (6.4.R1). The light blue lines represent the timeline, the dark blue lines indicate the time periods when the keyword appears, and the red lines represent the time periods when the keyword is burst (with high citation frequency).

Vitamin D (VD) is a fat-soluble vitamin with a wide range of biological effects. Its active form, 1α,25-dihydroxyvitamin D_3_ (1,25(OH)_2_D_3_), exerts its functions by binding to the VD receptor (VDR) [7]. The roles of VD extend beyond its well-known regulation of calcium and phosphorus metabolism and bone mineralization; it also plays a critical role in modulating immune responses and inflammation, regulating cell proliferation and differentiation, and inhibiting tumor growth [8,9,10,11]. Thus, VD is essential for maintaining homeostasis within the body. Recent studies have increasingly highlighted the close relationship between VD and the onset and progression of sepsis. We conducted a comprehensive keyword analysis through a bibliometric study, retrieving publications related to sepsis and VD/VDR from the Web of Science Core Collection (WoSCC) database from its inception to 2024 (Figure 1). High-frequency keywords such as critical illness, inflammation, infection, VD deficiency, and COVID-19 indicate that there is a growing interest in understanding the role of VD in immune modulation and the inflammatory response during critical illness. This also highlights the potential significance of VD in clinical outcomes for patients with sepsis.

VD has long been recognized as a key factor in regulating immune responses and influencing the outcomes of critical illnesses [12,13,14,15,16,17,18,19,20,21]. This review further updates the understanding of VDR, presents the roles of VD and VDR in sepsis, and describes mechanisms that contribute to their action. We also discuss the clinical implications and potential interventions of VD and its analogs in sepsis, providing valuable insights for clinical treatment of sepsis and the rational application of VD.

## 2. Clinical Evidence of VD Impact on Sepsis

### 2.1. VD Deficiency

VD deficiency (VDD) has emerged as a significant concern in critically ill patients, particularly those suffering from sepsis. A significant prevalence of low VD levels has been observed in critically ill patients, both pediatric and adult. In most pediatric ICUs (PICUs) globally, the rate of VDD varies from 30% to 70% [22]. Among adults, approximately 25% are at risk of VDD, while 8% face VD insufficiency [23]. A significant proportion of critically ill patients (ranging from 50% to 90%) admitted to the ICU exhibit low levels of VD [24,25,26,27]. Insufficient levels of VD impair immune function, disrupt hormone metabolism, and elevate the risk of various viral and bacterial infections and severe illnesses, ultimately leading to increased mortality [28]. Numerous studies have explored the prevalence and implications of VDD in sepsis patients, revealing a complex relationship between VD levels and clinical outcomes. Studies investigating VDD in patients with sepsis are summarized in Table 1. Researchers indicate that VDD or VD insufficiency is linked to an increased susceptibility to sepsis and poorer clinical outcomes, including greater sepsis severity, higher mortality rates, prolonged hospital and ICU stays, and negative correlations with lactate, CRP, acute physiology and chronic health evaluation II (APACHE II), and sequential organ failure assessment (SOFA) scores, as well as longer durations of mechanical ventilation [25,29,30,31,32,33,34,35,36,37,38,39,40,41]. However, some studies have found no significant connections between VD levels and the susceptibility to or severity of sepsis [42,43]. In pediatric populations, findings are also inconsistent. Some research shows that critically ill children and infants with sepsis may have lower levels of 25(OH)D and more severe VDD compared to those without sepsis [28,44,45,46], suggesting that lower 25(OH)D levels are associated with sepsis susceptibility and poor outcomes [46,47,48,49,50,51,52,53]. Conversely, other studies indicate that neither VD levels nor VDR polymorphisms are linked to sepsis [54,55].

### 2.2. VD Supplementation

While the evidence regarding the role of VD in sepsis treatment remains inconclusive, the potential benefits of supplementation in improving overall health outcomes cannot be overlooked. An overview of studies investigating VD supplementation in sepsis patients is presented in Table 2. Multiple randomized controlled trials (RCTs) indicate that high-dose VD safely elevates plasma 25(OH)D levels into the sufficient range without causing adverse effects such as hypercalcemia or hypercalciuria [59,60]. However, the benefits of VD supplementation for patients with sepsis remain debated. Some studies suggest that VD supplementation positively impacts the sepsis prognosis and infectious biomarkers, leading to better clinical outcomes, reduced mortality in mechanically ventilated patients, and shorter hospital stays [59,61,62,63,64,65]. A recent pharmacological evaluation investigating the clinical efficacy of VD in COVID-19 and long COVID has shown that extensive observational studies indicate a definitive relationship between low VD levels and the severity and mortality of COVID-19. It also reports beneficial effects of VD supplementation on COVID-19. Additionally, it has been highlighted that metformin may enhance VD receptor (VDR) sensitivity and improve treatment outcomes for both COVID-19 and long COVID [66]. Conversely, other research indicates that VD supplementation does not offer advantages over a placebo in terms of mortality or other nonfatal outcomes among critically ill patients with VDD [67,68,69,70]. In pediatric care, a case–control study found that maternal VD supplementation may enhance neonatal VD levels and reduce the risk of early-onset neonatal sepsis (EONS) [48]. Additionally, research involving children (<14 years) demonstrated that a single dose of 150,000 IU of cholecalciferol improved 25(OH)D levels and reduced the incidence of septic shock in those with VD deficiency and sepsis [71]. Another RCT in neonates with sepsis revealed that VD supplementation improved sepsis scores and lowered high levels of hs-CRP [72]. Furthermore, an RCT indicated that a dose of 400 IU of VD was sufficient to treat VD deficiency in most premature infants with late-onset sepsis [73].

### 2.3. Association of VDR Gene Polymorphism with the Risk of Sepsis

The relationship between VDR gene polymorphisms and susceptibility to sepsis has garnered increasing attention. The VDR gene is located on chromosome 12q13.1 and contains multiple single-nucleotide polymorphisms (SNPs), such as rs2228570, rs1544410, rs7975232, and rs731236 [74]. VDR gene polymorphisms have been shown to affect the expression and function of the VDR protein. For instance, the rs2228570 polymorphism is associated with changes in VDR protein levels in immune cells, and the rs1544410 and rs731236 polymorphisms are linked to variations in VDR plasma concentration [75]. Given that the VDR protein plays a crucial role in immune regulation, polymorphisms in the VDR gene may influence immune responses and susceptibility to diseases.

A recent case–control study indicated that the VDR gene may be a sepsis susceptibility gene in Chinese Han children [76]. The rs2228570 polymorphism in the VDR gene has been consistently associated with an increased risk of sepsis. A case–control study indicated that rs2228570 variants were more prevalent in sepsis patients compared to healthy controls, indicating a significant role in predisposing individuals to sepsis and septic shock [77]. A meta-analysis found that the rs2228570 locus, particularly the AA genotype, was linked to a higher susceptibility to sepsis (OR = 0.53, 95% CI = 0.30–0.91) [29]. A prospective observational study confirmed that the VDR rs2228570 polymorphism, particularly the CC genotype, appears to be associated with lower mortality in ICU patients [78]. The rs731236 polymorphism has shown mixed results. One study found an association between rs731236 SNP and neonatal sepsis, with specific genotypes (CT vs. CC + TT) being more prevalent in sepsis cases [79]. However, another meta-analysis did not find a significant correlation between rs731236 and sepsis risk [29]. The rs1544410 and rs7975232 polymorphisms have not shown a consistent association with sepsis across studies. Some analyses did not find significant correlations, suggesting that these polymorphisms may not play a major role in sepsis susceptibility [29,77].

The frequency of VDR polymorphisms may vary significantly among different ethnic groups, potentially influencing patients’ susceptibility to diseases. Researchers have investigated specific alleles and genotypes of VDR single-nucleotide polymorphisms, including rs731236, rs7975232, and rs1544410, within the Kazakh population, discovering that these exhibit unique prevalence patterns [80]. Further prospective studies and large-scale clinical trials are needed to explore the gene–environment interactions and the potential of using VDR polymorphisms as biomarkers for disease susceptibility and a therapeutic response.

## 3. Possible Mechanisms of the Association Between VD/VDR and Sepsis

### 3.1. Pathophysiological Process of Sepsis

#### 3.1.1. Innate Immunity and Inflammatory Mediators

Sepsis is a life-threatening syndrome characterized by organ dysfunction resulting from a dysregulated host response to infection and/or infectious agents [1]. Infection refers to the pathophysiological process of colonization and proliferation of pathogens, such as bacteria, fungi, viruses, protozoa, etc. Infectious agents are the fundamental structural molecules of these pathogens, including pathogen-associated molecular patterns (PAMPs) and damage-associated molecular patterns (DAMPs). PAMPs include bacterial lipopolysaccharides/endotoxins (LPSs), flagellin, genomic DNA, yeast polysaccharides, and single- or double-stranded RNA (ssRNA or dsRNA). DAMPs refer to intracellular material or molecules released from dead or damaged host cells, such as adenosine triphosphate (ATP) and mitochondrial DNA [81]. The first step in the host’s response to a pathogen is the activation of innate immune cells, primarily comprising macrophages, monocytes, neutrophils, and natural killer cells. During the infection process, invading pathogens, PAMPs, and DAMPs are recognized by pattern recognition receptors (PRRs) [82]. These PRRs, distributed in the immune cells, include toll-like receptors (TLRs), C-type lectin receptors, NOD-like receptors (nucleotide-binding oligomerization domain), RIG-I-like receptors (retinoic acid-inducible gene 1), etc. This recognition activates intracellular signal transduction pathways, resulting in the transcription and release of pro-inflammatory cytokines such as tumor necrosis factor-α (TNF-α), interleukin-1 (IL-1), and IL-6. Additionally, some PRRs, particularly the NOD-like receptor family, can form larger protein complexes known as inflammasomes, which play a critical role in producing vital cytokines such as IL-1β and IL-18, as well as caspases involved in programmed cell death. Pro-inflammatory cytokines promote the activation and proliferation of leukocytes, the activation of the complement system, the upregulation of endothelial adhesion molecules and chemokine expression, the production of tissue factors (TFs), and the induction of hepatic acute phase reactants. In sepsis, this immune response is exaggerated, leading to collateral damage and the death of host cells and tissues. The essence of sepsis lies in the fact that the PAMPs and DAMPs recognized by PRRs trigger immune cell activation, ultimately resulting in immune dysfunction.

#### 3.1.2. Immunosuppression

Although the precise timeline of immunological changes in sepsis remains inadequately characterized, accumulating evidence indicates that the progression of sepsis encompasses two distinct phases: an initial cytokine storm characterized by hyperinflammation, followed by a phase of immunosuppression marked by hypoinflammation [83]. These phases may coexist but predominate at different stages of the condition. The unregulated cytokine storm is primarily responsible for fatalities occurring within the initial days of sepsis, while immunosuppression is the leading cause of mortality in the later stages [84]. Notably, over 70% of patients die after the first three days of sepsis, likely due to heightened vulnerability to weakly virulent pathogens or opportunistic bacteria, reflecting the host’s inability to eliminate invading pathogens. This phase renders patients vulnerable to secondary infections and reactivation of latent infections, ultimately leading to increased mortality rates [2].

In immunosuppression patients, T-cell numbers decrease due to apoptosis and a reduced response to inflammatory cytokines. Autopsy studies of ICU patients who died from sepsis indicated a significant loss of CD4^+^ and CD8^+^ T cells, especially in lymphoid tissues like the spleen. Research also found reduced production of essential cytokines such as IL-6 and TNF-α in response to LPS [85]. Moreover, neutrophils in septic patients showed fewer chemokine receptors, leading to decreased chemotaxis to IL-8 [86]. Myeloid-derived suppressor cells (MDSCs) expand during sepsis and significantly inhibit both innate and adaptive immune responses, ultimately suppressing T-cell activation and proliferation, worsening the immunosuppressive environment [87]. A key feature of immunosuppression is the reprogramming of monocytes/macrophages. In septic patients, reprogrammed leukocytes may exhibit excessive release of anti-inflammatory factors and reduced MHC-II expression. Epigenetic regulation related to histone and DNA methylation plays a crucial role in this reprogramming of immune cells in sepsis. Sepsis-induced immunosuppression involves epigenetic changes in immune cells, leading to lasting immune function alterations in sepsis survivors. Identified epigenetic biomarkers may predict post-septic immunosuppression risk, providing potential strategies for improved management [88]. Additionally, studies indicate that autophagy in clearing dead and dysfunctional cells contributes to immunosuppression [89,90]. Furthermore, several studies highlight the close association between sepsis-induced immune suppression and the checkpoint regulator PD-1. Increased PD-1 expression can lead to T-cell apoptosis and higher mortality in septic patients [91]. Immunosuppression in sepsis thus provides a novel understanding of the disorder as well as a new therapeutic approach.

#### 3.1.3. Coagulation Disorder

In sepsis, inflammatory and hemostatic pathways intersect, activating both inflammatory and coagulation cascades. This disruption of the anticoagulant–coagulant balance leads to extensive thrombosis and can trigger severe disseminated intravascular coagulation (DIC). Coagulation abnormalities are closely linked to TF, expressed by endothelial cells, which activates FX and FIX when binding with FVIIa, amplifying coagulation through interactions with platelets and neutrophils [92]. Under pathological conditions, major anticoagulation pathways—antithrombin, TF pathway inhibitor, and the protein C system—are inhibited, increasing thrombosis risk [93]. Additionally, thrombin activates platelets, promoting neutrophil aggregation and releasing coagulation factors, enhancing coagulation further [94]. Research shows that coagulation factors like TF and fibrin can promote inflammation and activate a complement, which in turn can activate thrombin [95]. High-mobility group box 1 also plays a role in coagulation activation during sepsis. Recent studies indicate that Gasdermin D (GSDMD) induces pyroptosis, releasing TF and activating coagulation [96]. Targeting GSDMD may offer a new approach to inhibit abnormal coagulation in sepsis.

#### 3.1.4. Organ Dysfunction

The underlying mechanism of tissue and organ dysfunction in sepsis is decreased perfusion, resulting in reduced oxygen delivery and utilization by cells. This hypoperfusion is caused by cardiovascular dysfunction associated with sepsis [97]. The incidence of septic cardiomyopathy ranges from 18% to 60%, believed to be linked to circulating cytokines such as TNF-α and IL-1β, which can depress cardiac myocytes and disrupt mitochondrial function. The combination of hemodynamic changes and microvascular thrombosis can lead to inadequate perfusion of tissues and organs. Consequently, anaerobic glycolysis increases in cells, producing lactic acid. Additionally, reactive oxygen species (ROS) generated by the inflammatory response lead to mitochondrial dysfunction and decreased ATP levels. These factors cause cellular damage, resulting in widespread changes in tissues and organs that significantly contribute to the morbidity and mortality associated with sepsis [82].

### 3.2. VD/VDR

**Figure 2:**
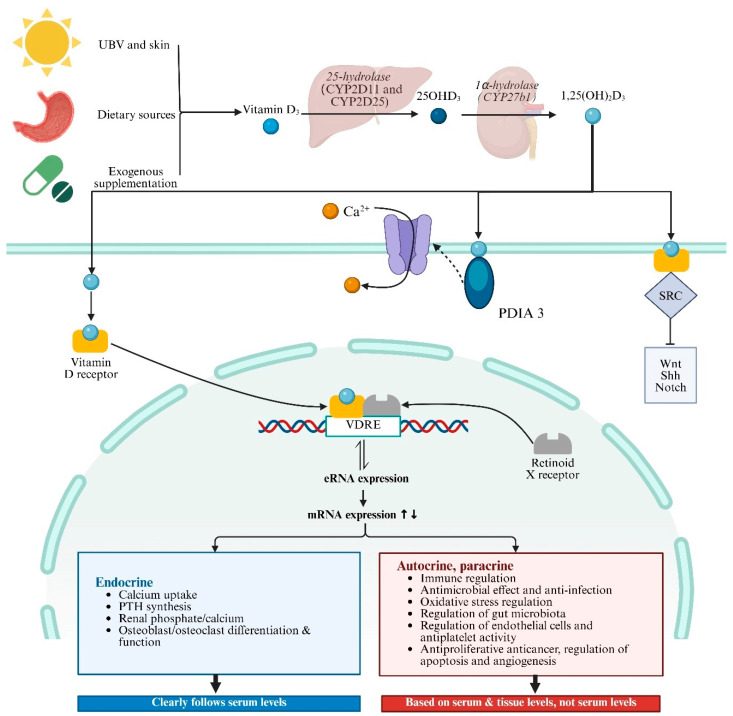
Vitamin D synthesis, signaling, and pathophysiological process. Abbreviations: UBV, ultraviolet radiation b; VDRE, vitamin D response elements; SRC, SRC proto-oncogene, non-receptor tyrosine kinase; PDIA3, protein disulfide isomerase family A member 3; PTH, parathyroid hormone; SRC, SRC proto-oncogene, non-receptor tyrosine kinase. ↑↓ indicates the impact of the upregulation or downregulation of mRNA expression levels on the following physiological functions.

VD is obtained in three main ways: skin synthesis from UV radiation acting on 7-dehydrocholesterol, dietary sources, and exogenous supplementation, and skin synthesis is the predominant method. VD has two main forms, Vitamin D_2_ (ergocalciferol) and Vitamin D_3_ (cholecalciferol), with Vitamin D_3_ being more effective in raising serum 25-hydroxyvitamin D levels [98]. Vitamin D_3_ enters the bloodstream and is transported to the liver by vitamin D binding protein (DBP), where enzymes like CYP2D11 and CYP2D25 hydroxylate it into 25-hydroxyvitamin D3 (25(OH)D_3_) [99]. 25(OH)D_3_ is the primary form of Vitamin D_3_ in plasma and serves as the main storage form in the liver. It is then further hydroxylated in the kidneys by 1α-hydroxylase to form 1,25(OH)_2_D_3_, which is critical for calcium homeostasis [98]. 1,25(OH)_2_D_3_ is the most biologically active form of VD, exerting its effects by binding to VDR in target cells (Figure 2). Global consensus recommendations on prevention and management of nutritional rickets define VD sufficiency as serum 25(OH)D_3_ levels above 50 nmol/L, deficiency as below 30 nmol/L, and insufficiency as between 30 and 50 nmol/L [100]. VD levels in populations are influenced by various factors including geographical location, seasonal variation, sunlight exposure, skin pigmentation, dietary sources, and supplement intake. Deficiency is particularly prevalent in high-latitude regions, during winter, and in overcast weather [101]. Currently, VD measurement methods include immunoassays and liquid chromatography–tandem mass spectrometry (LC-MS/MS). Immunoassays are popular due to their speed and convenience, but they cannot differentiate between 1,25(OH)_2_D_3_ and 25(OH)D_3_ and may have cross-reactivity issues [102]. In contrast, LC-MS/MS is highly sensitive and specific, regarded as the gold standard for measuring VD metabolites, though it is more time-consuming and costly [103,104].

The biological roles of VD in health and disease are linked to changes in the transcriptome mediated by 1,25(OH)_2_D_3_ in VDR-expressing cells (Figure 2) [105]. VDR, a member of the nuclear receptor superfamily, influences many genes across various cell types and tissues [106,107]. When 1,25(OH)_2_D_3_ binds to VDR, it undergoes a conformational change, allowing it to heterodimerize with the retinoid X receptor (RXR) [108,109]. This complex then travels to the nucleus, where it binds to VD response elements (VDREs) in gene promoters and recruits co-regulatory complexes to modulate transcription. The VDR–ligand complex regulates the expression of over 1000 genes through both activation and repression mechanisms [99,110,111,112]. VDR-mediated transcriptional regulation involves epigenetic modifications and chromatin reorganization. Enhancer RNAs (eRNAs), a class of non-coding RNAs, have emerged as potential VDR target genes. They facilitate chromatin looping and enhancer–promoter interactions, crucial for the activation of mRNA-encoding genes [113]. Genome-wide analyses have identified numerous VDR binding sites, with a significant number being unique to specific cellular contexts. These sites often contain DR3-type sequences, which are preferentially found at highly ligand-responsive VDR loci [114]. Besides the genomic pathway, there is increasing evidence that a subset of plasma-membrane-bound VDRs elicit rapid biological effects through nongenomic pathways [115,116]. Recent studies have identified rapid, nongenomic effects mediated by membrane-bound VDRs and protein disulfide isomerase family A member 3 (PDIA3) [117,118].

### 3.3. Immune Regulation

VDR is expressed in a variety of immune cells, including monocytes/macrophages, dendritic cells (DCs), T cells, B cells, etc., indicating its essential role in modulating immune responses. 1,25(OH)_2_D_3_ modulates the activation, proliferation, and differentiation of immune cells through the VDR expressed in these cells [119,120,121,122,123,124]. Additionally, immune and inflammatory cells can convert 25(OH)D_3_ into 1,25(OH)_2_D_3_ [125,126,127,128]. The expression of CYP27B1 is increased by immune stimuli (such as IFN-g and LPS) that activate the C/EBPb transcription factor, which binds to the CYP27B1 genes in both mice and humans [129].

#### 3.3.1. Monocytes/Macrophages

Monocytes are a type of white blood cell derived from the bone marrow, circulating in the blood. Macrophages are differentiated forms of monocytes found in tissues, possessing enhanced phagocytic and immune functions. 1,25(OH)_2_D_3_ stimulates the differentiation of monocytes to macrophages [130]. Activated macrophages, which function as antigen-presenting cells (APCs), release inflammatory mediators to attract various cell types to the site of inflammation for pathogen elimination. Macrophages are typically categorized into two types: M1 and M2. M1 macrophages generate pro-inflammatory mediators such as nitric oxide (NO), TNF-α, IL-23, IL-12, and IL-1β, facilitating pathogen clearance and promoting the polarization of T helper 1 (Th1) and Th17 cells. M2 macrophages produce IL-10, which possesses anti-inflammatory properties [131]. 1,25(OH)_2_D_3_ can influence macrophage polarization, promoting a shift towards an anti-inflammatory phenotype [132]. VDR activation promotes M2 macrophage polarization and then reduces liver ischemia and reperfusion injury. Autophagy is involved in this process by mediating the suppressor of cytokine signaling [133].

In monocytes and macrophages, the expression of the 1α-hydroxylase isoenzyme (CYP27B1) induces the production of active 1,25(OH)_2_D_3_, which activates the VDR and promotes cell adhesion and differentiation. Additionally, activated VDR induces the production of cathelicidin, thereby contributing to antimicrobial activity [134]. 1,25(OH)_2_D_3_ inhibits LPS-induced p38 activation and cytokine production in monocytes/macrophages by upregulating MKP-1 [135]. 1,25(OH)_2_D_3_ triggers IL-15-differentiated macrophages, inducing antimicrobial activity against intracellular *Mycobacterium tuberculosis* (Mtb) [136]. 1,25(OH)_2_D_3_ restores macrophage lysosome acidification to protect against Clostridioides difficile infection in mice [137]. 1,25(OH)_2_D_3_ suppresses LPS-induced inflammation by promoting negative feedback regulation of TLR signaling that targets microRNA-155-SOCS1 in macrophages [138]. VDR activation exhibits strong anti-inflammatory effects in mouse hepatic macrophages, including those isolated from diet-induced obesity livers, and mice with genetic loss of VDR developed spontaneous hepatic inflammation at 6 months of age [139]. Consistently with the data above, VDR KO mice are less responsive to LPS stimulation [140].

#### 3.3.2. DCs

DCs are pivotal in orchestrating immune responses, acting as APCs that bridge innate and adaptive immunity. DCs patrol tissues for pathogens, process foreign antigens, and present peptides to T cells, which then differentiate into effector cells. 1,25(OH)_2_D_3_ decreases the production of pro-inflammatory cytokines, such as IL-12 and TNF-α, while simultaneously enhancing the production of the anti-inflammatory cytokine IL-10 in DCs [141,142,143]. Furthermore, 1,25(OH)_2_D_3_ diminishes the expression of class II MHC and costimulatory molecules (CD40, CD80, CD86) in DCs. These DCs exhibit a limited capacity to induce T-cell proliferation and autoreactive T-cell activation, instead promoting the differentiation of regulatory T (Treg) cells [143,144,145,146]. 1,25(OH)_2_D_3_ suppresses IL-12 expression through the binding of the VDR/RXR complex to the NF-kB site within the IL-12p40 promoter [147]. Moreover, VD has been extensively proposed as a modulator of DCs towards tolerance, reducing their ability to stimulate effector T-cell generation while enhancing their potential to induce anti-inflammatory Treg cells [148,149,150]. DCs metabolize VD for the programming of T cells, and directly interact with DCs to influence their migration and their ability to instruct T cells, thereby initiating, fine-tuning, or attenuating immune responses [151].

#### 3.3.3. B Cells

B cells are essential components of the immune system, primarily responsible for producing antibodies that neutralize pathogens. They also present antigens to T cells, form memory B cells for long-term immunity, and secrete cytokines to regulate immune responses. In antibody-mediated autoimmune conditions, B cells are crucial for producing auto-reactive antibodies.

1,25(OH)_2_D_3_ has distinct effects at different stages of B-cell differentiation. It inhibits B-cell proliferation, immunoglobulin class switching, and antibody production while also inducing apoptosis [152,153]. 1,25(OH)_2_D_3_ directly regulates the homoeostasis of B cells, by promoting the apoptosis of immunoglobulin-secreting B cells and inhibiting the formation of plasma and memory cells [15,148,152]. The VDR directly and indirectly regulates IgE production in B cells. Through the VDR, VD is an environmental factor that helps to maintain low serum IgE responses [154].

Human B cells activated by CD40 and IL-4 signals can generate and secrete significant amounts of 1,25(OH)_2_D_3_. Moreover, 1,25(OH)_2_D_3_ enhances IL-10 expression in activated B cells by recruiting the VD receptor to the promoter of IL-10, and it can also increase IgE expression [127]. 1,25(OH)_2_D_3_ efficiently induces CCR10 expression in terminally differentiating human B cells in vitro. The human CCR10 promoter is cooperatively activated by Ets-1 and VDR in the presence of 1,25(OH)_2_D_3_ [155].

Furthermore, 1,25(OH)_2_D_3_ can influence the effect of B cells on regulating the activation and function of T cells. Naive T cells co-cultured with these B cells exhibited reduced expansion, decreasing cytokine production upon restimulation. The reduced CD86 expression on B cells was identified as a key mechanism, as T-cell activation and expansion were restored by anti-CD28 antibodies. 1,25(OH)_2_D_3_-primed B cells have a diminished capacity to activate T cells [156]. 1,25(OH)_2_D_3_ reduces the expression of CD74, which plays a role in the assembly and presentation of MHC-II molecules on the cell surface [157]. The impact of 1,25(OH)_2_D_3_ on the NF-kB pathway mediated CD40 activation to suppress the differentiation of B cells into plasma cells [155,158].

#### 3.3.4. T Cells

The expression level of VDR in naive T cells is low, rendering them insensitive to 1,25(OH)_2_D_3_. However, studies have shown that 1,25(OH)_2_D_3_ may directly regulate T-cell receptor (TCR) signaling and induce the expression of nuclear VDR and phospholipase C gamma 1 (PLC-γ1) through the MAPK p38 pathway [128,159]. Both VDR and PLC-γ1 play crucial roles in TCR signaling and T-cell activation [160]. T cells can differentiate into various subtypes, with 1,25(OH)_2_D_3_ exerting different effects on each subtype, influencing their differentiation, proliferation, and cytokine production [143,161,162]. TH lymphocytes are the specific cellular targets for the immunoinhibitory effects of 1,25(OH)_2_D_3_, which shifts T cells from a pro-inflammatory immune state to a more tolerant phenotype [163]. VDR is essential for the normal development and function of invariant natural killer T (iNKT) cells. The development of iNKT cells relies on the expression of functional nuclear VDR in the thymus. VDR KO mice exhibit impaired responses of iNKT cells to TCR stimulation [164].

1,25(OH)_2_D_3_ has been found to modulate the balance between pro-inflammatory and anti-inflammatory cytokines produced by T cells. This modulation is particularly important in the context of chronic inflammatory conditions, where an imbalance in cytokine production can lead to tissue damage and disease progression. 1,25(OH)_2_D_3_ inhibits the production of interferon gamma (IFN-γ), IL-2, IL-4, IL-9 (Th9), and IL-22 (Th22) in Th1 cells [120,162,165,166,167,168,169,170]. CD4^+^ T cells from VDR KO mice produce higher levels of IFN-γ and IL-17 compared to their wild-type counterparts [171]. Mechanistically, VDR/RXR can suppress IFN-γ expression by binding to a silencing region in the hIFN-γ promoter [172]. Additionally, 1,25(OH)_2_D_3_ inhibits IL-2 production in Th1 cells by directly interfering with the NFATp/AP-1 complex and sequestering nVDR from Runx1 through the VDR/RXR pathway [166].

1,25(OH)_2_D_3_ not only inhibits Th2 cell differentiation but also promotes the secretion of anti-inflammatory Th2 cytokines (such as IL-3, IL-4, IL-5, and IL-10) and GATA3 in Th2 cells without polarization [173,174]. During the in vitro polarization of naive CD62 ligand^+^ CD4^+^ T cells, calcitriol inhibits the production of the Th2 cytokine IL-4. Notably, VD exhibits its strongest inhibitory effect when administered from the beginning of the polarization process; however, adding VD after polarization significantly diminishes this effect. In activated CD62 ligand-CD4^+^ T cells, the inhibitory effect of 1,25(OH)_2_D_3_ on IL-4 production is absent. A potential mechanism underlying this is that, during Th2 differentiation, the VDR reduces IL-4 transcription [162,165].

Th17 cells are crucial for defending against certain pathogens, including *Helicobacter pylori*, Mtb, *Candida albicans*, *Klebsiella pneumoniae*, and *Staphylococcus aureus*, all of which are associated with inflammation and tissue damage [175]. In vitro, 1,25(OH)_2_D_3_ inhibits the production of Th17-related cytokines and transcription factors, such as IL-17A, IL-17F, RORC, and CCR6, thereby suppressing Th17 cell differentiation and activation [169,176]. 1,25(OH)_2_D_3_ also reduces the production of IFN-γ and TNF-α in CD8^+^ T cells [177]. 1,25(OH)_2_D_3_ significantly decreases the expansion of TCRγδ T cells, IFN-γ production, and CD25 expression in a dose-dependent manner. Additionally, it downregulates Akt and ERK signaling pathways and enhances antigen-induced cell death at higher concentrations [178].

Tregs, characterized as CD4^+^ CD25^+^ T cells, not only produce anti-inflammatory cytokines such as IL-10 and TGF-β but also downregulate the activity of macrophages, dendritic cells, and both CD4^+^ and CD8^+^ T cells. Tregs are essential for maintaining immune tolerance and preventing autoimmune diseases by helping to suppress excessive immune responses [179]. VD has been shown to influence the development, differentiation, and apoptosis of Tregs [180,181]. Forkhead box P3 (Foxp3) is a vital regulator of Treg development and function. 1,25(OH)_2_D_3_ modulates FOXP3 expression in Tregs through direct binding of the VDR to the FOXP3 gene [182]. In vivo administration of vitamin D3, along with the adoptive transfer of vitamin D3-induced IDO (+) immature/tolerogenic DCs, leads to a significant increase in Treg proportions in the lymph nodes of a rat model of multiple sclerosis [183]. Moreover, 1,25(OH)_2_D_3_ inhibits the production of pro-inflammatory cytokines, including IFN-γ, IL-17, and IL-21 in Tregs. Additionally, 1,25(OH)_2_D_3_ stimulates high levels of cytotoxic T-lymphocyte-associated protein 4 and FoxP3 expression, the latter of which requires the presence of IL-2 [180]. Furthermore, VD facilitates the polarization of CD4^+^ T cells towards Tregs or regulatory Th2 phenotypes by regulating the gene transcription of cytokines [184,185,186]. These two phenotypes are crucial for VD’s ability to suppress Th1-mediated autoimmune responses [161,186].

### 3.4. Antimicrobial Effect and Anti-Inflammation

#### 3.4.1. VD/VDR Induced Cathelicidin Pathway in Antimicrobial Effect

The 1,25(OH)_2_D_3_-VDR-RXR complex activates the transcription of genes encoding antimicrobial peptides (AMPs), which have direct antimicrobial activities against various pathogens via the formation of ion channels and increasing membrane permeability [187,188]. In addition to their antimicrobial roles, AMPs have significant regulatory effects on innate immunity and adaptive immunity, as well as on the aggregation and activation of immune cells and the expression of genes related to inflammatory factors [189]. AMPs directly regulated by VD signaling include human cationic AMP 18 (hCAP-18) and Defensin β2 (DEFB) [188]. The hCAP-18 is located in the phagolysosome with azurophil granule proteins, including serine protease. It converts hCAP-18 into the active AMP LL-37, which is a specific form of human cathelicidin [190]. The expression of LL-37 is significantly enhanced by 1,25(OH)_2_D_3_ in all assessed cell types, including epithelial cells, macrophages, and neutrophils, while the regulation of DEFB expression was less pronounced [191]. There has been a growing interest in designing new peptides based on AMPs [192]. Wang et al. identified the core antimicrobial region of LL-37 using 3D structural analysis, which can be function-dependent. They modified LL-37 into 17BIPHE2, which emerged as a stable, selective, and potent antimicrobial, antibiofilm, and anticancer peptide. Additionally, they proposed the application strategies of VD as a peptide-inducing factor [193].

AMPs acts as a vital regulator and effector of antimicrobial responses mediated by 1,25(OH)_2_D_3_. Gottlieb et al. developed a high-throughput cathelicidin assay using CRISPR/Cas9 edited human monocyte-macrophage cell lines, which serves as a new tool for evaluating VD-dependent antimicrobial responses [194]. A meta-analysis revealed a negative correlation between serum 25(OH)D_3_ levels and LL-37 in asthma patients [195]. Similarly, an RCT conducted in patients with acute respiratory infections also found a negative correlation between serum 25(OH)D_3_ levels and LL-37, suggesting that sufficient serum 25(OH)D levels may help prevent acute respiratory infections, potentially mediated by the induction of AMP production [196]. A pilot clinical trial involving 76 hospitalized patients with SARS-CoV-2 infection demonstrated the potential efficacy of VD supplementation in reducing the severity of COVID-19, with oral calcifediol reducing the need for intensive care unit (ICU) treatment [197]. Research by Mok et al. indicated that 1,25(OH)_2_D_3_ exhibits significant efficacy against SARS-CoV-2 in cell models, functioning by enhancing the expression of the AMP cathelicidin through the regulation of the VDR pathway [198].

LL-37 exhibits broad antimicrobial activity, which has been reported against Gram-positive and Gram-negative pathogenic bacteria, viruses, and fungi [187,199,200]. Moreover, LL-37 is considered an immunomodulator that exerts pro-inflammatory or anti-inflammatory effects depending on the specific cells, tissues, or microenvironments [201]. A substantial body of research indicates that VD exerts antibacterial effects against Mtb through the induction of antimicrobial peptides, highlighting its protective role against tuberculosis [202,203,204,205]. Studies have shown that the TLR family plays a crucial role in activating the expression of VDR and CYP27B1 in human macrophages, which in turn induces the activation of cathelicidin [134,206]. Cathelicidin-dependent autophagy is involved in inhibiting the replication of Mtb and HIV in co-infected macrophages [207]. Additionally, another study found that VD mediates antibacterial effects by regulating paracrine signaling in epithelial cells, including IL1R1 signaling and IL-1β-driven production of the antimicrobial peptide defensin beta 4 (DEFB4)/human beta-defensin 2, independent of 1,25(OH)_2_D_3_-stimulated autophagy in macrophages [208]. Fernandez et al. found that 1,25(OH)_2_D_3_ can limit the infection of monocyte-derived macrophages (MDMs) by the Zika virus (ZIKV). Transcriptional profiling revealed that VD can reduce the levels of pro-inflammatory cytokines and chemokines in MDMs, while enhancing the expression of degranulation-related genes like cathelicidin. Additionally, treatment with LL-37 was shown to decrease the proportion of ZIKV-infected MDMs [209]. Furthermore, Tang et al. discovered that the P2X7 receptor (P2X7R) plays an important role in the internalization of LL-37 by human macrophages, contributing to the intracellular clearance of bacteria [210].

#### 3.4.2. VD/VDR Regulating Autophagy Pathway

VD and VDR regulate autophagy through a different signaling pathway [211]. The role of the VD/VDR-antimicrobial peptide axis in regulating autophagy during infection has been extensively studied [191]. 1,25(OH)_2_D_3_/VDR-induced antimicrobial peptides activate autophagy in Mtb-infected monocytes, promoting the maturation of intracellular Mtb phagosomes and thereby clearing Mtb [202]. Antimicrobial peptides can be internalized into lysosomes, enhancing the bactericidal activity of target cells against *Staphylococcus aureus*, *Helicobacter pylori*, and *Salmonella* [210,212,213]. Mechanistically, VD-induced cathelicidin expression upregulates Beclin-1 and ATG5, thereby increasing autophagy levels. Moreover, the antimicrobial effects of 1,25(OH)_2_D_3_ and cathelicidin can be abrogated by autophagolysosome inhibitors [204,214]. Another study indicated that the combination of IL-12 and IL-18 triggered an antimicrobial response against Mtb through the activation of VDR, cathelicidin, and autophagy in p-38/MAPK- and STAT4-dependent pathways in macrophages and lung epithelial cells [215].

As a target gene of VDR, autophagy-related 16 like 1 (ATG16L1) serves as a crucial mediator in the VDR-regulated autophagy pathway. Studies in intestinal epithelial cells demonstrate that diminished VDR expression is associated with decreased ATG16L1 levels and compromised autophagy, consequently elevating the risk of inflammatory bowel disease [216]. In Paneth cell-specific VDR knockout mice, lysozyme production was markedly reduced in the Paneth cell. It demonstrated impaired antimicrobial capacity and diminished autophagic responses, highlighting VDR’s essential role in maintaining Paneth cell function and inflammatory regulation [217]. Interestingly, in LPS-tolerant macrophages, elevated VDR expression negatively regulates ATG16L1 transcription, resulting in suppressed autophagy and a reduced bacterial clearance capacity alongside diminished pro-inflammatory cytokine production [218]. These findings reveal that VDR’s transcriptional control of ATG16L1 exhibits cell-type specificity and varies according to immunological and inflammatory conditions.

In HK-2 cells, microtubule-associated protein 1 light chain 3 (LC3) interacts with VDR to facilitate its nuclear translocation and form activated VDR-RXR dimers, with the binding site distinct from the LC3-interacting region. LC3-RXR dimerization inhibits high glucose-induced activation and nuclear translocation [219]. Recently investigations demonstrate that P62 directly binds VDR through its nuclear receptor box motif, promoting VDR nuclear translocation and transcriptional activation of autophagy to suppress ferroptosis in the airway epithelium [220]. Lai et al. reported that VD reverses endothelial damage caused by ROS accumulation, mitophagy impairment, and inflammation [221]. Ma’s research identified E3 ubiquitin ligase Parkin as an interaction partner of VDR, promoting VDR degradation through P62-associated autophagolysosomal pathways [222]. In wrap-restraint stress-exposed rats, 1,25(OH)_2_D_3_ administration alleviates stress-induced colitis by enhancing AMPK activity and suppressing mTOR-STATS signaling to augment colonic autophagy [223]. In monosodium iodoacetate-induced osteoarthritis rat models, VD supplementation significantly ameliorated pain, synovial inflammation, and cartilage degradation by downregulating matrix metalloproteinase-13, IL-1β, and monocyte chemoattractant protein-1 expression while inducing autophagy activation [224].

#### 3.4.3. Modulation of VD/VDR on Inflammatory Cytokines

The inhibitory role of 1,25(OH)_2_D_3_ in inflammation has been widely studied in innate immune responses. VD has been found to reduce levels of pro-inflammatory cytokines (TNF-α and IL-6), while promoting the secretion of anti-inflammatory cytokines like IL-10 [225]. This shift in cytokine balance is crucial for mitigating the excessive inflammation that often accompanies sepsis, which can lead to organ dysfunction and increased mortality [226]. Cui et al. demonstrated that VDR-deficient microglia/macrophages exhibited a hyperinflammatory phenotype, marked by excessive secretion of TNF-α and IFN-γ, which further amplified endothelial CXCL10 production and T-cell infiltration, exacerbating brain injury. Conversely, VDR activation restrained these cytokines, preserving blood–brain barrier integrity and mitigating inflammatory damage [227]. 

1,25(OH)_2_D_3_ directly activates transcription of the *IL-1β* gene in human macrophages by promoting VDR binding to its promoter VDRE, thereby enhancing IL-1β production—a pivotal early cytokine in pathogen defense. This effect is amplified by NLRP3 inflammasome activation during Mtb infection. In a macrophage–epithelial co-culture model, 1,25D-driven IL-1β secretion reduced bacterial burden and improved macrophage survival via paracrine induction of epithelial AMPs. Additionally, 1,25D broadly amplifies cytokine/chemokine responses, including CCL3, CCL4, CCL8, and IL-8/CXCL8, highlighting its role in orchestrating innate immunity against infections [208]. In human peripheral blood mononuclear cells infected by Mtb, treatment with 1,25(OH)_2_D_3_ results in a dose-dependent inhibition of the pro-inflammatory cytokines IL-6, TNF-α, and IFN-γ [228]. Zhang et al. found that 1,25(OH)_2_D_3_ and 25(OH)_2_D_3_ dose-dependently inhibit LPS-induced p38 phosphorylation in human monocytes. This reduces IL-6 and TNF-α production via MKP-1 upregulation of pro-inflammatory cytokine production by targeting mitogen [135].

Through binding to the VDR, 1,25(OH)_2_D_3_ regulates innate immune responses of PRR signaling by activating transcription of the genes [229]. Interestingly, VD differentially regulates human innate cytokine responses to bacterial versus viral PRR stimuli. 1,25(OH)_2_D_3_ was found to inhibit inflammatory innate cytokine responses stimulated by bacterial PRR ligands but not by viral PRR ligands or infectious respiratory syncytial virus. This indicates a complex role of VD in immune modulation, particularly in bacterial infections [230].

The TLRs are the most prominent family of receptors triggering anti-inflammatory activity. TLR4 is a critical PRR that recognizes LPS from Gram-negative bacteria, triggering an inflammatory response. 1,25(OH)_2_D_3_ can negatively regulate the TLR4/myeloid differentiation primary response gene 88 (MyD88)/Toll-IL-1 resistance-domain-containing adapter-inducing interferon-β (TRIF) signaling pathway, thereby reducing the production of pro-inflammatory cytokines such as IL-6, IL-10, and TNF-α in monocytes/macrophages [231]. VD has been shown to reduce the expression of various TLRs, specifically TLR2, TLR4, and TLR9. Additionally, it inhibits the production of several pro-inflammatory cytokines in monocytes and macrophages, including IL-6, IL-23, TNF-α, inducible nitric oxide synthase (iNOS), IL-1, and various chemokines that recruit T cells [232]. Research has shown that VD can modulate cytokine production by enhancing the synergistic effect provided by NOD2 and TLR co-activation. This modulation results in increased levels of IL-10 and IL-23, while inhibiting IL-12p70 production [233]. VD/VDR signaling enhances innate immunity by directly upregulating the PRR NOD2/CARD15 in monocytes and epithelial cells. This induction facilitates synergistic activation of NF-κB upon stimulation by muramyl dipeptide (MDP), amplifying the expression of AMPs (cathelicidin antimicrobial peptide [CAMP] and DEFB4). Notably, this synergy is absent in macrophages from Crohn’s disease patients harboring non-functional NOD2 variants, underscoring the critical role of VD in bridging NOD2-mediated immune responses and inflammation regulation. These findings highlight VD’s dual role in modulating inflammatory pathways and antimicrobial defenses via VDR-dependent mechanisms [234]. 

VDR suppresses NF-ĸB-driven inflammatory responses in the gut through direct interactions with NF-ĸB and its upstream kinase-inhibitory kappa B kinase beta (IKKβ)IKKβ, thereby mitigating intestinal inflammation [235]. In macrophages, VD-activated VDR binds to the NF-κB p50 subunit, inhibiting its colocalization with Kruppel-like factor 5 and thereby suppressing LPS-induced cellular proliferation [236]. Studies have shown that the VDR negatively regulates bacteria-stimulated NF-κB activity by interacting with NF-κB p65, such as reducing its phosphorylation and nuclear translocation [237,238]. Interaction of VDR with NF-ĸB p65 impedes its migration into the nucleus, thereby dampening inflammatory signaling pathways. VD treatment can prevent LPS-induced placental inflammation by promoting dimerization of VDR with NF-ĸB p65 [239]. Artesunate interacts with VDR and disrupts its association with NF-κB p65 in LPS-tolerant macrophages, facilitating NF-κB p65 nuclear translocation. This activated the transcription of NF-κB p65 target genes such as pro-inflammatory cytokine-related genes [218].

#### 3.4.4. Modulation of VD/VDR on microRNA

The VD/VDR also participates in the regulation of the immune system through the modulation of microRNAs, which is closely associated with systemic or local inflammation. Karkein et al. reported that 1,25(OH)_2_D_3_ regulates the NF-κB signaling pathway by influencing miR-146a, miR-150, and miR-155 in adipocytes, thereby limiting inflammation [240]. Another study indicated that 1,25(OH)_2_D_3_ modulates the expression of inflammation-related miR-146a-5p and miR-155-5p during dengue virus type 2 infection in MDMs, thus affecting the inflammatory response [241]. The in vivo and in vitro studies also suggest that VD downregulates miR-155, thereby influencing the NF-κB signaling pathway and reducing the p65 subunit [240,242]. Interestingly, research has found that inflammation conversely downregulates VDR expression in epithelial cells by inducing miR-346 targeting VDR, thereby impairing mucosal barrier function [243]. Other studies suggest that VD/VDR regulates the immune system through microRNAs to assist in the treatment of sepsis. Shang et al. discovered that inhibiting miR-874-5p expression increases VDR expression, reduces NLRP3 expression, decreases caspase-1 activation and IL-1β secretion, and alleviates pyroptosis and inflammatory responses, thereby protecting from intestinal barrier damage in sepsis—all of which can be reversed by downregulating VDR [244]. Sepsis-induced acute lung injury (ALI) endangers patients’ lives, and controlling ALI helps improve the survival rate of sepsis patients. Ahmad et al. found that vitamin D-related microRNAs regulate the endoplasmic reticulum (ER) stress pathway in ALI to improve clinical outcomes. In a mouse model of sepsis combined with ALI, researchers found that VD improves the lung tissue structure, neutrophil infiltration, endothelial barrier, and reduces the upregulation of ER stress markers activating transcription factor 6 (ATF6) and C/EBP homologous protein induced by CLP and LPS. VD enhances the expression of miR-149-5p, which inhibits the ER-specific ATF6 inflammatory pathway in LPS-stimulated macrophages and improves pulmonary edema in mice [245]. These pieces of evidence indicate that the mechanism by which VD/VDR regulates the inflammatory level in sepsis involves its influence on microRNAs.

### 3.5. Oxidative Stress Regulation

Oxidative stress, characterized by excessive reactive oxygen species (ROS) and lipid peroxidation, plays a pivotal role in sepsis pathogenesis, driving tissue damage, mitochondrial dysfunction, and dysregulated immune responses. VD and VDR modulate oxidative stress through multiple mechanisms, particularly by targeting ferroptosis, and key redox signaling pathways. In a study on cardiomyocytes, Chen et al. demonstrated that organic oxidants, such as tert-butyl hydroperoxide, induce ferroptosis via glutathione depletion, glutathione peroxidase 4 (GPX4) degradation, and mitochondrial iron overload through the Bach1/heme oxygenase 1 (HO-1) pathway. While hydrogen peroxide (H_2_O_2_) induced milder oxidative stress, organic oxidants promoted robust lipid peroxidation and iron accumulation, illustrating the differential impacts of oxidative species on cell death mechanisms. This highlights the critical role of VD in mitigating ferroptosis: VD activates antioxidant pathways that scavenge ROS and stabilize mitochondrial function, countering excessive lipid peroxidation and iron-dependent cytotoxicity [246].

Cai et al. investigated neonatal hypoxic–ischemic encephalopathy and found that VD suppresses ferroptosis by activating the Nrf2/HO-1 pathway. VD treatment increased glutathione (GSH) levels, reduced the levels of malondialdehyde (MDA) and ROS, and upregulated GPX4, a key enzyme in lipid peroxide reduction [247]. Similarly, Li et al. showed that in aging mice, VD alleviates ferroptosis in hippocampal neurons by enhancing nuclear factor erythroid 2–related factor 2 (Nrf2) signaling, reducing the levels of intracellular iron and ROS, and improving mitochondrial morphology [248]. These findings suggest VD-mediated Nrf2 activation as a universal mechanism to counteract oxidative stress-induced lipid peroxidation and iron overload, which are critical in sepsis-associated organ injury. Hu et al. explored VDR’s role in cisplatin-induced acute kidney injury (AKI), revealing that VDR activation upregulates GPX4 transcription, inhibits lipid peroxidation (measured by 4HNE and MDA), and attenuates ferroptosis. This mechanism is particularly relevant to sepsis-induced AKI, where oxidative stress and renal tubular cell death contribute to morbidity. The study identified GPX4 as a direct target of VDR, linking VDR signaling to the regulation of ferroptotic pathways in renal protection [249]. In neuroinflammatory contexts, Ribeiro et al. showed that VD supplementation rescues aberrant NF-κB pathway activation in Mecp2 mutant mice. NF-κB, a key regulator of inflammation and oxidative stress, is hyperactivated in sepsis, driving pro-inflammatory cytokine production. VD-mediated inhibition of NF-κB reduces pro-inflammatory gene expression, potentially dampening the excessive immune response and oxidative tissue damage seen in severe sepsis [250].

### 3.6. Regulation of Gut Microbiota

Emerging evidence highlights a bidirectional regulatory interplay between VD/VDR signaling and gut microbiome dynamics, particularly in inflammatory bowel disease (IBD). Clinical and preclinical studies demonstrate that the VD status modulates the microbial composition, while supplementation enhances diversity via VDR-mediated antimicrobial and immune pathways. Ismailova et al. observed a higher prevalence of VD deficiency in patients with Crohn’s disease. Interestingly, Crohn’s disease patients with higher VD levels exhibited lower risks of disease relapse [251]. In addition, both British and American guidelines suggest that for IBD patients, VD levels should be checked in a timely manner. If VD deficiency occurs, VD supplementation should be carried out promptly [252,253]. A clear association exists between VD and IBD, particularly Crohn’s disease, which demonstrate significant connections with gut microbiome alterations.

Wang et al. found that human VDR gene variations significantly affect the composition of the gut microbiota. The study enrolled 1812 individuals from Germany and, after comprehensively controlling for dietary and non-genetic parameters, identified significant genome-wide associations between overall microbial variation/individual taxa and multiple genetic loci (including the VDR gene). Integrated with preclinical studies, they found that both non-genetic and genetic factors each accounted for approximately 10% of gut microbiota variation [254]. Luthold et al. reported higher *Prevotella* but lower *Haemophilus* and *Veillonella* levels in fecal samples from individuals with a higher VD intake [255]. A systematic meta-analysis encompassing 25 clinical trials demonstrated that an elevated VD intake enhances gut microbiota diversity, inducing compositional shifts marked by altered alpha/beta diversity and increased microbial species richness [256]. Fabisiak et al. demonstrated that colonic mucosal expression of VDR and the antimicrobial peptide CAMP (LL-37) was significantly elevated in ulcerative colitis patients compared to healthy controls. VD maintains intestinal barrier integrity, regulates autophagy and apoptosis, and modulates gut microbiota composition through VDR signaling. VD deficiency is associated with exacerbated inflammation and microbial dysbiosis in IBD patients, while VD supplementation may improve gut microbial homeostasis by regulating VDR and antimicrobial peptide expression [257]. Kanhere et al. point out that weekly high-dose VD3 supplementation affects the intestinal and respiratory microbiota of patients with cystic fibrosis [258]. Interestingly, one study has also found that the gut microbiota is related to the absorption or metabolism of VD [259]. Aggeletopoulou et al. demonstrated in animal studies that VD modifies the gut microbial composition. VD deficiency or VDR knockout alters microbial flora. VDR knockout mice showed increased *Bacteroides* levels, while dietary restriction and CYP27B1 deficiency mice showed increased *Firmicutes* and *Proteobacteria* levels [260]. These pieces of evidence suggest that there may be a complex relationship between VD and gut microbiota in the human body.

### 3.7. Regulation of Endothelial Cells and Antithrombotic Activity

Sepsis involves endothelial dysfunction, where VD and VDR play crucial roles in modulating endothelial cell (EC) functions critical for immune balance and vascular integrity. Amersfoot et al. point out that some ECs serve as “immunomodulatory ECs (IMECs)”, which act as an early defender in sepsis. Those IMECs express VDR and regulate immune cell recruitment via adhesion molecules (e.g., vascular cell adhesion protein 1, intercellular adhesion molecule 1) and cytokines [261]. 1,25(OH)_2_D_3_ activates VDR in ECs to suppress pro-inflammatory pathways like NF-κB, reducing cytokines (IL-6, TNF-α) and improving endothelial nitric oxide synthase (eNOS) activity, which mitigates sepsis-induced vascular leakage and oxidative stress [262]. Liu et al. demonstrated that VD reduces apoptosis, promotes migration, and enhances the viability of ECs, while decreasing TNF-α-induced protein 8-like 1 (TIPE1) expression under high-glucose conditions. Their findings suggest that VD counteracts the detrimental effects of high glucose on ECs by suppressing TIPE1 expression, further indicating that VD supplementation may mitigate microvascular damage [263]. Song et al.’s knockout of the VDR of mice’s retina ECs exhibited impaired retinal vascular regeneration, increased inflammatory responses, and compromised EC regeneration compared to wild-type controls (VDR^+/+^). It showed that maintaining the physiological functionality of VDR in ECs is essential for preserving normal endothelial cell function [264]. Some evidence suggests that VD/VDR could benefit endothelial progenitor cells (EPCs). In vitro studies and animal models indicated that activation of VDR signaling enhances EPC functionality, and it may improve EC activity and stimulate angiogenic processes [265]. VD/VDR regulates EC adhesion, inflammatory responses, and repair mechanisms, potentially mitigating sepsis-induced endothelial dysfunction and immune dysregulation.

VD/VDR primarily exerts antithrombotic effects through antiplatelet and anticoagulant mechanisms [266]. Research on antiplatelet activity, concerning VD/VDR in sepsis, is limited but highlights key interactions. Cox proposed that platelets play a dual role in infectious diseases, exhibiting both protective and pathogenic effects. In sepsis, platelets release antimicrobial peptides (e.g., platelet factor 4 [PF4]), enhance monocyte bactericidal activity, and form microthrombi to limit pathogen dissemination. However, excessive platelet activation may contribute to DIC and organ failure. Some studies suggest that antiplatelet therapy may improve clinical outcomes [267]. A clinical study involving 503 patients requiring dual antiplatelet therapy (DAPT) demonstrated an association between VD and high residual platelet reactivity (HRPR). Specifically, the study revealed that lower VD levels were significantly correlated with increased adenosine diphosphate (ADP)-mediated platelet reactivity (*p* < 0.05). Researchers suggest that VD may potentiate the therapeutic efficacy of antiplatelet medications [268]. The United Kingdom’s clinical guidelines indicate that VD exerts antithrombotic effects through three primary mechanisms. Notably, 50% of thrombotic antiphospholipid syndrome (APS) patients were found to have VD deficiency. Importantly, APS patients with confirmed VD deficiency demonstrated a significantly higher incidence of thrombosis compared to those without deficiency (77% vs. 53%, respectively) [269]. VD/VDR is also associated with anticoagulation. Çakır et al. found in a study involving 201 patients with atrial fibrillation that low 25(OH)D_3_ levels were correlated with the occurrence of left atrial thrombus in patients taking non-vitamin K antagonist oral anticoagulants. They suggested that VD supplementation or deficiency might influence the efficacy of anticoagulant drugs, warranting further investigation [270]. Khansari et al. reported similar findings, indicating that VD levels affect the therapeutic efficacy of warfarin [271]. An animal study demonstrated that VD supplementation was associated with downregulation of TNF-α-induced TF expression and NF-κB signaling, significant attenuation of PAR-2 expression, restoration of VDR levels, and enhanced TF pathway inhibitor (TFPI) expression. TFPI is an anticoagulant protein that functions as a dual coagulation inhibitor by binding to TF/factor VIIa and factor Xa [272,273].

## 4. Critical Knowledge Gaps and Future Perspectives

### 4.1. Limitations and Gaps in Current Research

Current evidence on the association between VD deficiency and sepsis susceptibility remains inconclusive, plagued by heterogeneous study designs, divergent diagnostic thresholds for VD insufficiency, and inconsistent patient populations. Observational studies report correlations between hypovitaminosis D and an increased sepsis incidence or mortality, yet RCTs evaluating VD supplementation in critically ill patients have yielded conflicting results, failing to establish a consensus on its therapeutic efficacy. For example, while preclinical models demonstrate that 1,25(OH)_2_D_3_ enhances AMP production and modulates cytokine profiles in immune cells, these findings are not consistently replicated in human sepsis trials. This translational gap likely stems from multifactorial challenges, including the complexity of sepsis pathophysiology, interindividual variability in VD metabolism, and suboptimal timing or dosing of supplementation in clinical settings.

Moreover, methodological inconsistencies further confound interpretation. Serum 25(OH)_2_D_3_—the widely accepted biomarker of VD status—exhibits limitations in critically ill populations. Hemodilution from fluid resuscitation can artificially lower 25(OH)D levels by up to 35%, while immune cell-specific activation of VD may render serum measurements inadequate for assessing tissue-level bioactivity [274]. Numerous studies have demonstrated significant variations in VD content across different tissues, as well as substantial differences among various VD forms. Lipkie et al. reported marked differences in 25(OH)D_3_ concentrations across rat soft tissues, adipose tissue, and liver [275]. Similarly, Fu et al. found distinct concentrations of 25(OH)D_3_ and 1,25(OH)_2_D_3_ in human brain tissue compared to other body tissues [276]. Máčová et al. highlighted several challenges in current VD testing methodologies, including sample type selection and the choice of immunoassays versus chromatography or mass spectrometry. Notably, there is no globally standardized VD measurement protocol. The researchers emphasized the need for improved antibody specificity and standardized mass spectrometry procedures [277]. Nevertheless, the global management of VD shows significant variations. Across different regions, local guidelines demonstrate substantial disparities in the defined concentration thresholds for VD deficiency, insufficiency, sufficiency, and toxicity (Table 3) [278,279]. Additionally, our understanding of VD toxicity and side effects remains limited. For patients with hepatic or renal impairment (the two major organs involved in VD metabolism), sepsis, and other critically ill patients, the definitions of VD standard are poorly established. The potential side effects of VD supplementation in these populations are also unclear [277,278]. There are no widely accepted standards regarding the timing and dosage of VD supplementation for these patient groups.

Animal models, though instrumental in elucidating mechanistic pathways (e.g., VDR-mediated transcriptional regulation of IL-1β or NLRP3 inflammasome modulation), poorly replicate the immunometabolic dysregulation of human sepsis. These discrepancies underscore the need for standardized protocols to define VD sufficiency in sepsis, stratified RCTs targeting deficient subpopulations, and advanced models that bridge the gap between preclinical promise and clinical reality.

### 4.2. Future Directions

Future studies can prioritize elucidating the physiological role of VD/VDR in sepsis, particularly in resolving controversies surrounding its immunomodulatory effects. This requires systematic investigations into its dynamic states and functions across different sepsis phases, especially sepsis-induced immunosuppression. Our research team has made some strides in understanding VDR’s role during sepsis-associated immunosuppression. Our findings highlight VDR as a bidirectional immunoregulatory checkpoint in the sepsis pathogenesis and suggest that pharmacological modulation of the VDR–autophagy–NF-κB axis could represent a promising therapeutic strategy [218]. Our recent work uncovered a previously unrecognized form of VDR-membrane-bound VDR (mVDR) on macrophages, which plays a pivotal role in LPS tolerance formation [116]. Building on these findings, future studies may benefit from exploring the temporal dynamics of VDR activation in human sepsis, clarifying its stage-dependent functions, and investigating potential therapeutic synergies through integration of VD/VDR modulation with established immunoadjuvant strategies.

Emerging evidence underscores the need for population-specific VD thresholds. Genetic polymorphisms in VDR (e.g., *FokI* and *BsmI* variants) and ethnic disparities in VD metabolism necessitate tailored approaches [77,80]. For instance, populations in high-latitude regions with limited sunlight exposure, such as Finland, exhibit higher incidences of autoimmune diseases like type 1 diabetes—conditions potentially modifiable by VD optimization [280,281,282]. Similarly, infants, elderly patients, and those with renal/hepatic impairment require distinct supplementation protocols to mitigate toxicity risks while maximizing immune benefits [28,44,45,46]. Future guidelines can integrate genetic, geographic, and comorbidity factors to define precision thresholds for deficiency and sufficiency.

To bridge this gap, stratified clinical trials targeting VD-deficient subpopulations, such as critically ill adults and children, are essential. The ongoing VITdALIZE and VITdALIZE-KIDS trials exemplify this approach. The VITdALIZE study (NCT03188796) is an international (Austria, Germany, Belgium, Switzerland, and UK), multicenter (more than 30 sites), placebo-controlled, double-blind, phase 3 randomized trial, which aims to evaluate the outcomes (such as 28-day morality) of VD supplementation on the prognosis of critically ill adult patients with VD deficiency [283]. Subsequently, in September 2024, VITdALIZE-KIDS (NCT03742505) was designed to assess the effect of VD supplementation on outcomes in children (>37 weeks, <18 years), using a study method similar to the VITDALIZE study [284]. These two studies may provide insights into the clinical response to VD supplementation in different critically ill patients, and both have infection-related end points that warrant continued attention.

VD analogs have been identified in over 3000 species. The majority of these analogs have not yet progressed to clinical trials or received regulatory approval for use as drugs or dietary supplements [285]. Beyond classical roles in bone health, VD analogs show promise in modulating immune dysregulation. Tacalcitol, a synthetic VD analog, has demonstrated efficacy in autoimmune diseases (psoriasis and vitiligo) without disrupting calcium and phosphorus homeostasis [286,287]. The pharmacological actions of Tacalcitol primarily involve the inhibition of DNA synthesis and the suppression of proliferation in human epidermal cells derived from psoriasis lesions [286,288]. Tacalcitol also influences the immune system, particularly in acute lymphoblastic leukemia (ALL) B cells, such as those expressing CD27, CD24, CD38, and CD23 [289]. Similarly, Oxcalcitriol, approved for secondary hyperparathyroidism, has shown potential in autoimmune diseases like rheumatoid arthritis (RA), reducing joint inflammation with fewer hypercalcemic side effects compared to calcitriol [290,291]. Oxcalcitriol also exhibits renoprotective effects in both diabetic nephropathy and acute kidney injury by attenuating inflammatory mediators (IL-16, TLR-4, IFN-γ), reducing apoptosis/fibrosis, and enhancing autophagy to promote cell survival [292,293]. Preclinical studies of Maxacalcitol reveal its ability to attenuate psoriasiform inflammation by upregulating regulatory T cells and downregulating IL-23/IL-17 pathways, outperforming corticosteroids in reducing MHC Class II-driven immune infiltration [294]. These findings suggest VD analogs could be repurposed for sepsis-associated hyperinflammation, particularly given their pleiotropic effects on autophagy, apoptosis, and TLR4 signaling—key pathways dysregulated in septic organ failure.

Research investigating VD/VDR–microRNA interactions in sepsis warrants significant expansion. Current investigations predominantly focus on localized complications such as intestinal barrier dysfunction and acute lung injury [244,245], and a definitive empirical delineation of their holistic impact on sepsis pathophysiology concurrently lacks clinical validation. Future trials can also incorporate tissue-specific biomarkers (e.g., LL-37 levels, VDR activity) and advanced technologies (single-cell sequencing, organoid models) to refine VD’s therapeutic window. Combining dynamic serum profiling with tissue biopsies or immune cell assays may better predict clinical responses. Additionally, leveraging AI-driven pharmacokinetic models could optimize dosing schedules for high-risk populations, ensuring efficacy while minimizing adverse events.

## 5. Conclusions

VD/VDR signaling represents a promising yet underexplored avenue for sepsis management. VD/VDR may serve as a promising bidirectional immunomodulator, capable of addressing sepsis’s hyperinflammatory and immunosuppressive phases. Achieving clinical translation requires bridging mechanistic insights with precision medicine approaches, ensuring that VD-based therapies are timed, dosed, and targeted to align with sepsis’s dynamic pathophysiology. By addressing current knowledge gaps and leveraging novel analogs and technologies, VD/VDR modulation could evolve into a cornerstone of personalized sepsis care, offering dual benefits as an immune enhancer and anti-inflammatory agent across all disease stages.

## Figures and Tables

**Table 1:** Summary of studies on vitamin D deficiency in sepsis patients.

Year	Country	Period	Study Population	Study Design	Main Findings	Reference
2024	China	As of 2023	UK Biobank and FinnGen consortium	Mendelian randomization	No significant connections were observed for VD.	[42]
2023	-	As of 2023	5 studies	Systematic review and meta-analysis	Patients with sepsis have lower 25(OH)D levels, and the VDR Fok I polymorphism is closely associated with the susceptibility to sepsis.	[29]
2023	China	2023	UK Biobank data in 2021	Mendelian randomization	No evidence of a causal association between vitamins and sepsis risk from a genetic perspective.	[43]
2023	Republic of Korea	2019–2020	129 sepsis patients	Prospective cohort study	Severe VDD can independently affect poor prognosis related to sepsis.	[30]
2023	India	2013–2014	96 sepsis patients, age > 18 years	Retrospective cohort study	Measurement of VD at the time of admission may be a possible indicator for prediction of sepsis mortality and hospital and ICU length of stay for ICU-admitted patients with sepsis.	[31]
2023	India	2022	80 sepsis patients, aged 18–60 years (40 case group and 40 control group)	Case–control study	A decrease in VD levels correlates with the incidence of mortality in sepsis patients.	[32]
2022	Egypt	2019–2020	50 children with sepsis, aged from 1 month to 13 years old with community-acquired infections within the first 48 h of admission to the ICU	Case–control study	In children admitted to pediatric ICU, neither VD levels nor VDR polymorphisms were associated with sepsis.	[54]
2021	-	2014–2019	18 cohort and case–control studies	Systematic review and meta-analysis	Low levels of VD both in the cord blood and maternal blood were significantly associated with neonatal sepsis.	[47]
2021	-	As of 2020	27 studies with 17 case–control studies and 10 cohort studies	Systematic review and meta-analysis	Critically ill infants and children with sepsis could have a lower 25(OH)D level and severe VDD compared to those without sepsis.	[28]
2021	India	2018–2019	62 infants in each group of cases and control	Case–control study	VD insufficiency is significantly associated with EONS. VDD significantly increases risk of EONS.	[48]
2021	The Republic of Türkiye	2019–2020	148 patients diagnosed with sepsis	Prospective observational study	VDR levels were associated with high 28-day mortality and negatively correlated with lactate, CRP, APACHE II, and SOFA scores and disease severity in patients with sepsis.	[33]
2020	-	As of 2020	16 studies with 2382 children	Systematic review and meta-analysis	VDD in children might have an independent relationship with higher sepsis, pediatric risk of mortality III score, length of hospital stay, and duration of mechanical ventilation.	[49]
2020	-	As of 2019	8 studies with 1736 patients	Systematic review and meta-analysis	Severe VDD may be independently associated with increased mortality in adult patients with sepsis.	[34]
2020	-	As of 2019	23 studies with 4451 children, 2500 children with VDD	Systematic review and meta-analysis	VDD in children may have an independent relationship with up to 2.24-fold risk of sepsis and acute and critical care unit mortality.	[50]
2019	-	As of 2018	52 studies, 7434 children with acute or critical conditions	Systematic review and meta-analysis	Meta-analysis of mortality (18 cohort studies, 2463 total individuals) showed increased risk of death in 25(OH)D-deficient children (OR 1.81, 95% CI 1.24 to 2.64, *p* = 0.002, I2 = 25.7%, *p* = 0.153).	[53]
2019	-	As of 2019	18 articles involving 2987 critically ill children	Systematic review and meta-analysis	A significant association was only observed in very highly developed countries between VDD and risk of sepsis [OR (95% CIs): 2.65 (1.30, 5.41)] and ventilation support requirement [OR (95% CIs): 1.35 (1.03, 1.77)].	[52]
2019	-	2011–2017	13 studies, comprising 975 patients and 770 control participants	Systematic review and meta-analysis	The association between VDD and sepsis was significant, with an odds ratio (OR) = 1.13 (95% CI, 1.18 to 1.50, *p* < 0.05) in children.	[56]
2019	The Republic of Türkiye	2017–2018	51 term infants in sepsis group and 56 term infants in control group	Prospective cohort study	The number of the newborns with VDD was significantly higher in the sepsis group (n = 31, 60.8%) than in the control group (n = 30, 53.6%; *p* = 0.00), corresponding to significantly lower levels of VD in the sepsis group (11 ± 5.5 ng/mL vs. 13.8 ± 10.6 ng/mL; *p* = 0.012). Similarly, maternal VD level was significantly lower in the sepsis group than in the control group (10.8 ± 5.6 ng/mL vs. 14.9 ± 10 ng/mL; *p* = 0.001).	[44]
2019	The Slovak Republic	2019	32 patients, admitted to an intensive care unit with both SIRS and sepsis	Retrospective cohort study	VDD predisposed to the development of sepsis, negatively correlated with CRP, presepsin, sTREM-1, and SOFA scores and their levels associated with both 7- and 28-day survival of patients.	[35]
2018	Republic of Korea	2013–2017	188 very-low-birth-weight infants	Retrospective cohort study	The results showed that 79.8% of preterm infants in this study had VD deficiency at birth. Low VD status was associated with respiratory morbidity.	[57]
2017	The Republic of Türkiye	2013	117 premature infants with gestational age of <37 weeks were enrolled	Prospective observational study	There was no significant relationship between the cord blood VD levels and the risk of neonatal sepsis in premature infants.	[55]
2016	Rome	2013–2014	107 patients with sepsis/septic	Retrospective cohort study	In critical septic patients, extremely low VD levels on admission may be a major determinant of clinical outcomes.	[36]
2015	-	2000–2014	10 observational studies, involving 33,810 participants	Systematic review and meta-analysis	VDD was associated with an increased susceptibility of sepsis.	[37]
2015	The Republic of Türkiye	2011–2012	40 newborns with EONS, 43 controls	Case–control study	Cord-blood 25(OH)D levels of neonates with EONS were significantly lower than those of the healthy controls, and a low level of cord-blood VD was found to be associated with an increased risk of EONS.	[45]
2015	Ireland	2012–2013	120 children with suspected sepsis admitted to the PICU and 30 pediatric controls	Prospective cohort study	Children admitted to the PICU with suspected sepsis had lower 25(OH)D than controls, and inadequate 25(OH)D status was associated with confirmed sepsis and poor outcomes.	[51]
2015	The Republic of Türkiye	2012	50 term infants with clinical and laboratory findings of early-onset sepsis EOS (study group) and 50 healthy infants with no signs of clinical/laboratory infection (control group)	Prospective observational study	Maternal and neonatal 25(OH)D levels (22.2/8.6 ng/mL, respectively) in the study group were significantly lower than those of the control group (36.2/19 ng/mL, respectively, *p* < 0.001). A positive correlation was detected between maternal and neonatal 25(OH)D levels. Severe VDD was significantly more common in the sepsis group.	[46]
2014	Austria	2008–2010	655 surgical and nonsurgical critically ill patients with available 25(OH) D levels hospitalized	Prospective observational study	Low 25(OH)D status is significantly associated with mortality in the critically ill.	[38]
2014	United States of America	2006–2011	121 patients, 25-hydroxyvitamin D levels measured within 30 days, admission for severe sepsis or septic shock	Retrospective cohort study	Patients deficient in VD within 30 days of hospital admission for severe sepsis or septic shock may be at increased risk for all-cause 30-day mortality.	[39]
2012	The State of Israel	2008–2009	107 patients admitted to ICUs and internal medicine wards	Prospective observational study	Low vitamin D levels are common among patients admitted to ICU. Longer survival times were observed among vitamin D-sufficient patients. Vitamin D concentration may be either a biomarker of survival or a co-factor.	[58]
2012	Canada	2002–2003	196 patients, age ≥ 18 years, expected to stay more than 24 h in the ICU and were enrolled within the first 24 h of ICU admission	Prospective cohort study	This study demonstrates significant decreases in VD status over the duration of the patient’s ICU stay. Low levels of VD are associated with longer time to ICU discharge alive and a trend toward increased risk of ICU-acquired infection.	[40]
2011	United States of America	1998–2009	2399 patients, age ≥ 18 years, pre-admission 25(OH)D was categorized as deficiency in 25(OH)D (≤15 ng/mL), insufficiency (16–29 ng/mL), and sufficiency (≥30 ng/mL)	Retrospective cohort study	Deficiency of 25(OH)D prior to hospital admission is a significant predictor of short- and long-term all-cause patient mortality and blood culture positivity in a critically ill patient population.	[25]
2011	United States of America	2011	81 patients, age ≥ 18 years, with suspected infection	Prospective observational study	VD insufficiency was associated with higher sepsis severity in emergency department patients hospitalized for suspected infection.	[41]

Abbreviations: VD, vitamin D; 25(OH)D, 25-hydroxyvitamin D; VDR, vitamin D receptor; VDD, vitamin D deficiency; UK, United Kingdom; ICU, intensive care unit; PICU, pediatric intensive care unit; OR, odds ratio; CI, confidence interval; CRP, C reaction protein; APACHE II, acute physiology and chronic health evaluation II; sTREM-1, soluble triggering receptor expressed on myeloid cells-1; SOFA, sequential organ failure assessment.

**Table 2:** Summary of studies on vitamin D supplementation in patients with sepsis.

Year	Country	Period	Study Population	Study Design	Main Findings	Reference
2024	The Islamic Republic of Iran	2017–2018	28 SIRS-positive patients, 14 patients received intravenous calcitriol daily for 3 days (intervention group), 14 patients (control group)	RCT	The administration of intravenous calcitriol could reduce the levels of procalcitonin but did not have a significant effect on presepsin. The ICU length of stay and 28-day mortality did not differ significantly either between the two arms of the study.	[61]
2023	China	2008–2019	3539 patients with sepsis (>18 years), VD supplementation group (n = 3224), non-VD supplementation group (n = 315)	Retrospective cohort study	VD supplementation during an ICU stay was associated with improved prognosis in patients with sepsis, as evidenced by lower in-hospital, 28-day, and 90-day mortality rates and lower disease severity-related scores, but showed no influence on the length of stay in the hospital or ICU.	[65]
2023	United States of America	2008–2019	19,816 adult patients with suspected infection in ICU, VD group (n = 714), non-VD group (n = 19,102)	Retrospective cohort study	VD supplementation demonstrated a lower risk of sepsis (odds ratio 0.46; 95% CI 0.35–0.60; *p* < 0.001) and a lower risk of new mechanical ventilation requirement (odds ratio 0.70; 95% CI 0.53–0.92; *p* = 0.01), but no significant difference in the risk of 28-day mortality was observed (hazard ratio 1.02; 95% CI 0.77–1.35; *p* = 0.89).	[64]
2022	Egypt	2020–2021	116 patients (group 1, n = 58: orally administered alfacalcidol 1 μg/day; group 2, n = 58: intramuscularly administered cholecalciferol 200,000 IU)	RCT	High-dose VD was considered a promising treatment in the suppression of cytokine storms among COVID-19 patients and was associated with better clinical improvement and fewer adverse outcomes compared to low-dose VD.	[62]
2021	India	2018–2019	62 infants in each group of cases and control	Case–control study	Risk of EONS increased 8 times in neonates with 25(OH)D level < 30 ng/mL (odds ratio = 8.2; 95% confidence interval [CI]: 3.08–21.82; *p* = 0.000). The 25OH-D level was significantly lower in EONS group than control group. Optimal cut-off for 25(OH)D was 25 ng/mL to predict EONS with a sensitivity and specificity of 88.7% and 79%, respectively (area under the curve: 0.84; 95% CI: 0.76–0.92; *p* = 0.000).	[48]
2020	China	2020	Children aged ≤ 14 years with VDD and sepsis to receive one dose of 150,000 IU of cholecalciferol (treatment group, n = 60) or placebo (control group, n = 60)	RCT	Ang-II, IL-6, and TNF-a concentrations were all reduced after vitamin D supplementation. Furthermore, the cv-SOFA score (1.76 ± 0.8 vs. 2.3 ± 1.1) and incidence of septic shock (7% vs. 20%) were lower in the treatment group than in the control group. The duration of ventilation and mortality rates did not differ between two groups.	[71]
2020	Egypt	2017–2019	60 neonates with sepsis, group I: 30 neonates with sepsis who received antibiotic only, group II: 30 neonates with sepsis who received antibiotic therapy and VD	RCT	Serum 25(OH)D levels of neonates with the early-onset neonatal sepsis were significantly lower than the healthy controls. VD supplementation improved sepsis score and decrease high levels of hs-CRP.	[72]
2019	United States of America	2014–2016	Preterm infants with gestational age (GA) ≥ 28 weeks with late-onset sepsis LOS, subjects were randomly assigned to receive 400 or 800 IU/day of vitamin D3. 25 infants in each group	RCT	Serum pro-inflammatory cytokines IL-6 and TNF-α concentrations decreased at 1 week and at discharge in both groups without differences between groups. A dose of 400 IU of VD was adequate to treat VDD in the majority of premature infants with LOS. The 2 dosing regimens did not differ in clinical or biochemical changes.	[73]
2019	-	As of 2018	52 trials with a total of 75,454 participants	Systematic review and meta-analysis	VD supplementation alone was not associated with all-cause mortality in adults compared with placebo or no treatment. VD supplementation reduced the risk of cancer death by 15%.	[67]
2019	The Islamic Republic of Iran	2019	Mechanically ventilated adult patients, placebo group (n = 18), VD group (n = 22)	RCT	High-dose VD could reduce mortality in mechanically ventilated patients. Despite decrease of 10 days in duration of mechanical ventilation, the difference was not statistically significant.	[63]
2019	United States of America	2017–2018	1360 patients, placebo group (n = 540), VD group (n = 530)	RCT	Early administration of high-dose enteral vitamin D3 did not provide an advantage over placebo with respect to 90-day mortality or other, nonfatal outcomes among critically ill, VDD patients.	[68]
2016	United States of America	2011–2014	ICU adult patients, placebo group (n = 10), 250,000 IU VD3 group (n = 10), 500,000 IU VD3 group (n = 11)	RCT	High-dose VD3 safely increased plasma 25(OH)D concentrations into the sufficient range and was associated with decreased hospital length of stay without altering other clinical outcomes.	[59]
2014	United States of America	2013	67 critically ill patients with severe sepsis or septic shock, 36 received calcitriol (2 μg intravenously), 31 received placebo	RCT	Calcitriol-treated patients had higher cathelicidin (*p* = 0.04) and IL-10 (*p* = 0.03) mRNA expression than placebo-treated patients 24 h after study drug administration. Plasma cytokine levels (IL-10, IL-6, TNF-α, IL-1β, and IL-2) and urinary kidney injury markers were similar in calcitriol-versus placebo-treated patients (*p* > 0.05 for all comparisons). Calcitriol had no effect on clinical outcomes nor were any adverse effects observed.	[69]
2014	Austria	2010–2012	492 critically ill adult white patients with VDD (20ng/mL), VD group (n = 249), placebo group (n = 243)	RCT	Among critically ill patients with VDD, administration of high-dose VD compared with placebo did not reduce hospital length of stay, hospital mortality, or 6-month mortality. Lower hospital mortality was observed in the severe VDD subgroup.	[70]
2011	Austria	2009–2010	25 adult patients with VDD and an expected stay in the ICU > 48 h, VD group (n = 12), placebo group (n = 13)	RCT	A single oral ultra-high dose of cholecalciferol corrects VDD within 2 days in most patients without causing adverse effects like hypercalcemia or hypercalciuria.	[60]

Abbreviations: VD, vitamin D; 25(OH)D, 25-hydroxyvitamin D; VDD, vitamin D deficiency; SIRS, systemic inflammatory response syndrome; EONS, early-onset neonatal sepsis; LOS, late-onset sepsis; IL-10, interleukin-10; TNF-α, tumor necrosis factor-α; hs-CRP, high-sensitivity C reaction protein; IU, international unit; ICU, intensive care unit; SOFA, sequential organ failure assessment; OR, odds ratio; CI, confidence interval.

**Table 3:** Vitamin D guidelines in different regions.

	Deficiency *	Insufficiency *	Sufficiency *	Toxicity *
Global	<50~25	30–75	50–250	>250
Europe	<50	50–75	50–225	>500
USA	<50	50–75	>75	>150
China	<30	30–50	≥50	
Brazil	<50	50–74	75–250	
Japan	<50	50–75	>75	
Australia	<29	30–49	≥50	
Gulf Region	<50	50–75	>75	

* All values are detected in the blood serum and reported as nmol/L.

## Abbreviations

VD	vitamin D
VDR	vitamin D receptor
MODS	multiple organ dysfunction syndrome
ICUs	intensive care units
1,25(OH)_2_D_3_	1α,25-dihydroxyvitamin D_3_
VDD	vitamin D deficiency
PICUs	pediatric intensive care units
CRP	c-reactive protein
APACHE II	acute physiology and chronic health evaluation II
SOFA	sequential organ failure assessment
EONS	early-onset neonatal sepsis
RCT	randomized controlled trial
SNPs	single nucleotide polymorphisms
LPS	lipopolysaccharides (endotoxins)
PAMPs	pathogen-associated molecular patterns
DAMPs	damage-associated molecular patterns
PRRs	pattern recognition receptors
TLRs	toll-like receptors
NOD	nucleotide-binding oligomerization domain
RIG-I	retinoic acid-inducible gene 1
TNF-α	tumor necrosis factor-α
IL-1	interleukin-1
MDSCs	myeloid-derived suppressor cells
MHC-II	major histocompatibility complex class II
PD-1	programmed cell death protein 1
DIC	disseminated intravascular coagulation
TF	tissue factor
FX	coagulation factors
GSDMD	gasdermin D
ROS	reactive oxygen species
ATP	adenosine triphosphate
DBP	vitamin D binding protein
LC-MS/MS	liquid chromatography-tandem mass spectrometry
RXR	retinoid X receptor
VDREs	vitamin D response elements
eRNAs	enhancer RNAs
PDIA3	protein disulfide isomerase family A member 3
DC(s)	dendritic cell(s)
IFN-g	interferon-gamma
APC	antigen-presenting cell
NO	nitric oxide
Th1:	T helper 1 cell
CYP27B1	25-hydroxyvitamin D3 1-alpha-hydroxylase (enzyme)
TCR	T cell receptor
PLC-γ1	phospholipase C gamma 1
iNKT	invariant natural killer T cell
IFN-γ	interferon gamma
Treg	regulatory T cell
Foxp3	forkhead box P3
AMP	antimicrobial peptide
hCAP-18	human cationic antimicrobial peptide 18
DEFB	defensin beta
LL-37	cathelicidin antimicrobial peptide (Active form of hCAP-18)
Mtb	*mycobacterium tuberculosis*
ZIKV	zika virus
ATG16L1	autophagy related 16 like 1
LC3	microtubule associated protein 1 light chain 3 (Autophagy marker)
MyD88	myeloid differentiation primary response 88
TRIF	TIR-domain-containing adapter-inducing interferon-β
IKKβ	inhibitor of nuclear factor kappa B kinase subunit beta
GSH	glutathione
MDA	malondialdehyde
GPX4	glutathione peroxidase 4
Nrf2	nuclear factor erythroid 2–related factor 2
HO-1	heme oxygenase 1
IBD	inflammatory bowel disease
CAMP	cathelicidin antimicrobial peptide
DEFB4	defensin beta 4
IMECs	immunomodulatory endothelial cells
eNOS	endothelial nitric oxide synthase
TIPE1	TNF-α-induced protein 8-like 1
EPCs	endothelial progenitor cells
PF4	platelet factor 4
DAPT	dual antiplatelet therapy
ADP	adenosine diphosphate
APS	antiphospholipid syndrome
HRPR	high residual platelet reactivity
WoSCC	Web of Science Core Collection
TF	tissue factor
TFPI	tissue factor pathway inhibitor
MDMs	monocyte-derived macrophages
ER	endoplasmic reticulum
ALI	acute lung injury
ATF6	activating transcription factor 6
